# A Case Report of Discoid Lupus Erythematosus Mimicking Skin Infection

**DOI:** 10.3390/reports9010004

**Published:** 2025-12-22

**Authors:** Zhenya Stoyanova, Elitsa Hinkova, Filka Georgieva, Hristo Popov, George Stoyanov

**Affiliations:** 1 Department of Infectious Diseases, Parasitology and Dermatovenereology, Medical University—Varna, 9000 Varna, Bulgaria; 2 Faculty of Medicine, Medical University—Varna, 9000 Varna, Bulgaria; 3 Department of General and Clinical Pathology, Forensic Medicine and Deontology, Medical University—Varna,9000 Varna, Bulgaria; 4 Department of Pathology, Multiprofile Hospital for Active Treatment, 9700 Shumen, Bulgaria

**Keywords:** discoid lupus erythematosus, bacterial infection, tuberculosis, fungal infection, induration, antiphospholipid syndrome

## Abstract

**Background and Clinical Significance**: Cutaneous lupus erythematosus (CLE) is an autoimmune condition characterized by a wide range of cutaneous manifestations, classified into three major subtypes—chronic (CCLE), subacute (SCLE), and acute (ACLE)—based on clinical morphology and lesion duration. Discoid lupus erythematosus (DLE), the most common form of CCLE, predominantly affects sun-exposed areas and presents as erythematous macules that progress to well-demarcated, disc-shaped plaques. If left untreated, DLE may lead to scarring and permanent alopecia. Diagnosis is primarily clinical, with skin biopsy performed when indicated. Management includes photoprotection and topical corticosteroids, with systemic immunosuppressive therapy reserved for severe cases. **Case Presentation**: We report a case of a 38-year-old female patient presenting with confluent lesions with indurated borders and multiple pustules, initially raising suspicion of cutaneous infection. A broad differential diagnosis was considered, including fungal and bacterial infections, demodicosis, and cutaneous tuberculosis, all of which were excluded through comprehensive clinical and laboratory investigations. Ultimately, DLE was diagnosed based on serologic and histopathologic findings. During the course of immunosuppressive therapy, her condition deteriorated, and she developed pulmonary tuberculosis. **Conclusions**: The presented case underlines the rarity and broad differential diagnosis of DLE as well as the possibility of complications.

## 1. Introduction and Clinical Significance

Lupus erythematosus is a chronic autoimmune disease with a variety of clinical manifestations that affect both the skin and internal organs with multiple differential diagnoses [1,2,3,4,5,6,7,8]. From a dermatological point of view, two main forms are distinguished—systemic lupus erythematosus (SLE) and cutaneous lupus erythematosus (CLE) [7,8,9,10,11,12]. Cutaneous lupus erythematosus encompasses several clinical subtypes—acute, subacute, and chronic forms—among which the most common is discoid lupus erythematosus (DLE), a prototypical manifestation of chronic cutaneous lupus [7,8,9,10,11,12,13,14,15]. DLE is characterized by chronic, well-circumscribed erythematous and hyperkeratotic plaques that most often affect sun-exposed areas such as the face, ears, scalp, and neckline. These lesions can leave permanent scars and cicatricial changes, which emphasize the importance of early diagnosis and appropriate treatment [10,11,12,13,14,15,16,17,18]. In Bulgaria, there is a lack of accurate epidemiological data on the prevalence of DLE; however, clinical practice suggests a number of cases, warranting increased attention among specialists in dermatology and general medicine. In both Europe and the United States, the reported incidence of isolated CLE ranges from 4.0 to 4.3 cases per 100,000 individuals, which is slightly higher than the SLE incidence rate, estimated at approximately 3 cases per 100,000 individuals. DLE accounts for 50% to 85% of all CLE cases. It exhibits a marked female predominance, occurring two to three times more frequently in women compared to men. While DLE can manifest across all age groups, its onset is most commonly observed between the ages of 20 and 40 years [19,20,21]. While DLE is the dominant form of CLE, it remains a rare condition that is difficult to recognize at initial presentation by non-dermatologists [1,2,3,4,5,6]. Initial misdiagnosis of the condition leads to extensive progression and the development of further complications.

In addition to the difficulty in recognition and diagnosis, DLE and other forms of lupus also present a further challenge—treatment and follow-up—as the immunosuppressive treatment can produce complications of its own [17,18]. Herein, we present a clinical and morphological case report of DLE with an atypical clinical presentation and a complication against the background of the immunosuppressive treatment—pulmonary tuberculosis.

## 2. Case Presentation

A 38-year-old woman with no evidence of concomitant chronic diseases and past medical history notable for two spontaneous abortions, followed by a single full-term delivery complicated by postpartum anemia requiring blood transfusion, presented to our department with complaints of skin changes starting two months prior.

**Figure 1:**
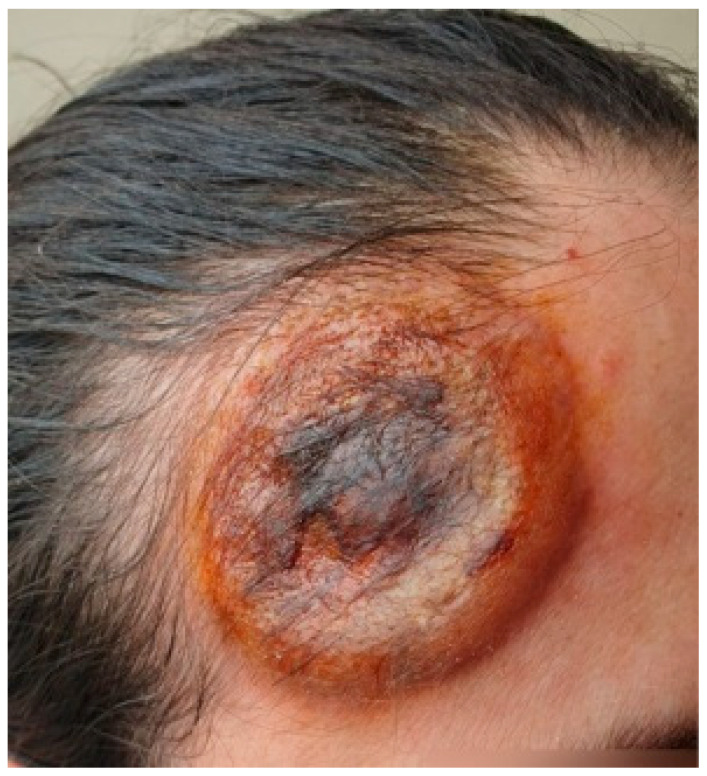
Solitary lesion with a well-demarcated indurated border and centrally located hemorrhagic crusts.

The first skin complaints noted by the patient were two “pustules” (as described and defined by the patient) on her forehead. Despite antibiotic treatment with clindamycin (600 mg orally, three times a day, for 7 days), the lesions progressed—according to the patient, they transformed into “dense blisters”. Later, local treatment was performed by a maxillofacial surgeon, who applied an occlusive dressing twice daily with povidone-iodine solution for 5 days. As a result, the skin changes demonstrated progressive increases in both periphery and height. During a focused interview, the patient reported photosensitivity (Figure 1).

Given a clinical suspicion of cutaneous tuberculosis, a Mantoux test was performed, yielding a borderline negative result. A skin biopsy revealed findings consistent with “fungal dermatitis” without identification of a specific pathogen. The patient consulted various specialists who prescribed diverse systemic treatments over time, including amoxiclav/clavulonic acid, itraconazole, and betamethasone. Due to a lack of therapeutic response, a parasitological skin examination was performed, in which commensal Demodex folliculorum was identified on a native preparation. Systemic and topical metronidazole treatment was initiated but proved ineffective.

**Figure 2:**
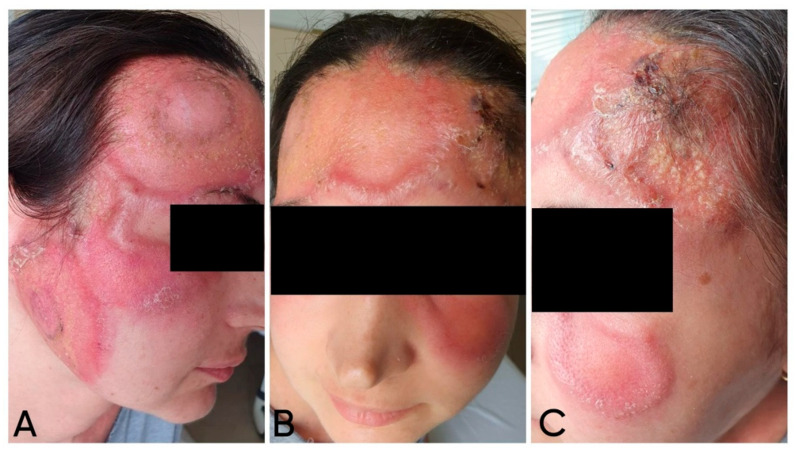
Gross view of the cutaneous changes upon presentation: (**A**) right lateral view—erythematous plaques with an active indurated peripheral border; (**B**) frontal view—pityriasisiform scaling; (**C**) left lateral view—pustules and crusts.

Upon admission to our department for diagnostic clarification, physical examination showed a polymorphic rash involving the central facial area, with near-symmetrical extension to the zygomatic regions. Multiple large erythematous plaques were observed, featuring an active, indurated peripheral border and centrally erythematous skin with scattered pityriasis-like scaling. In some areas, the plaques were confluent, covered with numerous pustules and brownish crusts (Figure 2).

Complete blood count and differential count revealed erythropenia and lymphocytopenia, decreased monocytes, and leukocytes at the lower limit of the reference range (Table 1).

**Figure 3:**
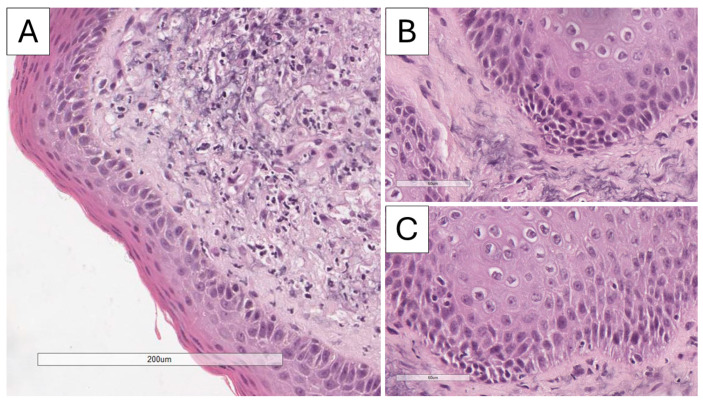
Histopathology of the lesion, H&E stain: (**A**) Epithelial atrophy with flattening of the rete ridges, original magnification 200×; (**B**) basal layer vacuolation, original magnification 400×; (**C**) basal layer vacuolation and apoptosis, original magnification 400×.

**Figure 4:**
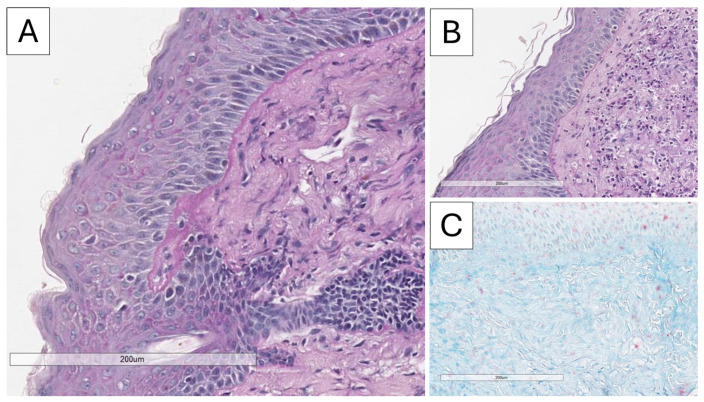
Histopathology of the lesion, PAS and Alcian blue stain. (**A**,**B**) Thickened PAS-positive basal membrane, original magnification 200×; (**C**) Alcian blue-positive dermal mucin accumulation, original magnification 200×.

**Figure 5:**
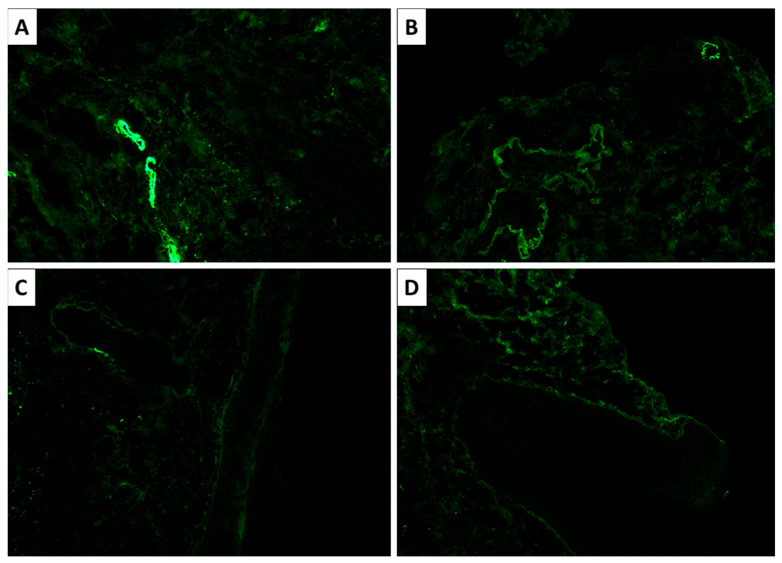
Immunofluorescence of the lesion. (**A**,**B**) C3 deposition in vascular walls, original magnification 100×; (**C**,**D**) discrete IgM deposition dermoepidermal deposition, original magnification 100×.

Histopathological and immunohistochemical analysis confirmed the suspected diagnosis of discoid lupus erythematosus. Microscopy revealed atrophic stratified squamous keratinizing epithelium, with flattened rete ridges, basal layer vacuolization, focal apoptosis, and a thickened basement membrane (PAS-positive) (Figure 3 and Figure 4). The dermis exhibited perivascular and periadnexal lymphoplasmacytic infiltrates, admixed with neutrophils, areas of fibrinoid necrosis of collagen fibers, and basophilic material accumulation (Alcian blue positive; consistent with mucin deposition) (Figure 4). Additional findings included pigment incontinence and follicular plugging. Direct immunofluorescence demonstrated C3 deposition in vascular walls, along with discrete dermoepidermal IgM deposition (Figure 5).

**Figure 6:**
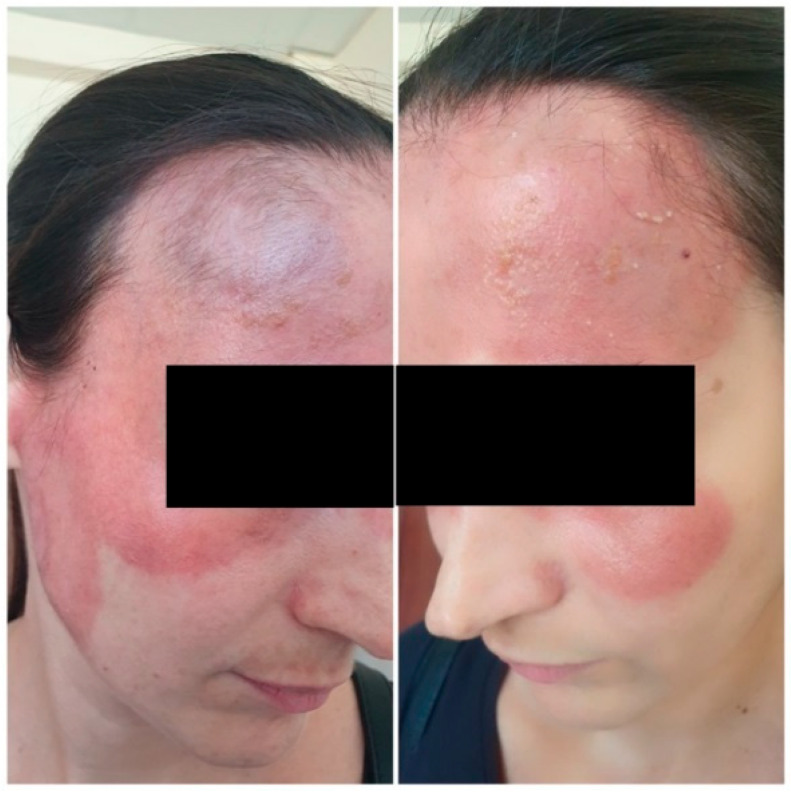
Gross view of post-treatment skin changes: (**Right**) lateral view—slightly erythematous, hyperpigmented skin color; (**left**) lateral view—scant amount of yellowish crusts.

Due to the pyodermic appearance of the lesions, initial antimicrobial therapy was started with ciprofloxacin, clindamycin, and fluconazole. After receiving the laboratory results, given the severity of the skin lesions and the extremely impaired quality of life of the patient, in accordance with the guideline for the treatment of cutaneous lupus, treatment was initiated with 40 mg of methylprednisolone intravenously, topical corticosteroid cream twice/day, and intensive photoprotection [18]. During hospitalization, a marked improvement was observed, including the absorption and drying of the purulent lesions, fading of the erythema, and resolution of edema, along with post-lesional hyperpigmentation (Figure 6). For home treatment, the patient was prescribed 16 mg of methylprednisolone daily, a topical corticosteroid cream, and high-factor sun protection. No further infection workup or antibiotic treatment was prescribed for home care due to the significant improvement in inflammatory parameters upon discharge, as seen in Table 1.

Given the diagnosed condition, the patient was referred to the Rheumatology Clinic for further monitoring and treatment. Detailed medical history, clinical evaluation, and laboratory tests did not show evidence of SLE and confirmed the diagnosis of a CCLE—DLE. Aberrations were noted in elevated antiphospholipid antibodies, suggesting the presence of antiphospholipid syndrome; however, no specific treatment was initiated, as not all criteria for the syndrome were met (lack of thrombotic events and organ-specific changes such as heart disease, despite her history of two prior miscarriages, as they occurred after the 10th gestational week) (Table 2). Due to the low antibody titers, lack of other major criteria, and the potential for antibody elevation in the context of inflammation and some treatments, no follow-up titers were tested.

The immunosuppressive therapy was continued with hydroxychloroquine 200 mg (one tablet in the evening); methylprednisolone 4 mg, which was reduced to three tablets in the morning, gradually decreasing by ½ tablet daily over 7 days until reaching a maintenance dose of two tablets daily; and acetylsalicylic acid 75 mg (one tablet in the evening).

One month later, the patient was readmitted to the Rheumatology Clinic due to worsening of her skin condition—redness and edema in the left lacrimal area. Gradually, the edema subsided, and a “red spot” with scaling developed, accompanied by intense itching. She also reported pronounced hair growth in the area of the old lesions. A dermatologic consultation revealed a solitary erythematous-squamous lesion in the left lacrimal area, oval-shaped, approximately 2–3 cm in size. A fungal infection was suspected, possibly triggered by the ongoing treatment with methylprednisolone (two tablets daily) and hydroxychloroquine, or a new flare-up of the underlying disease. The patient was prescribed phenoconazole nitrate cream. Immunosuppressive systemic therapy continued as initially prescribed by the rheumatologists.

During follow-up, with the patient’s general condition deteriorating, she was repeatedly hospitalized in the Rheumatology Clinic and the Internal Medicine Clinic. In her latest hospitalization, she was diagnosed with pulmonary fibrosis in an inflammatory flare, and pulmonary tuberculosis was confirmed via PCR test of sputum. Follow-up Ziehl–Neelsen stain of the original biopsy specimens did not show evidence of acid-fast bacilli. Antituberculosis treatment was initiated, and two years after the initial presentation of the cutaneous lesions, the patient remains under regular follow-up, with only discrete residual hyperpigmentation on the forehead. No further complications suggestive of APS have occurred since the initial workup.

## 3. Discussion

Cutaneous lupus erythematosus (CLE) is an autoimmune, inflammatory disease of the skin, characterized by a wide range of lesions, including malar rash, discoid plaques, psoriasiform or annular lesions of a polycyclic type, and scarring alopecia [7,10]. Based on clinical morphology, histopathology, and lesion duration, CLE is categorized into three major subtypes: acute (ACLE), subacute (SCLE), and chronic (CCLE) [8].

Discoid lupus erythematosus (DLE) is the most common form of CCLE, comprising 50–80% of chronic cases. Lesions are usually confined to sun-exposed areas such as the face, ears, scalp, and neck. The disease typically begins with erythematous macules or papules that evolve into well-defined, disc-shaped plaques with adherent scales. A hallmark feature is follicular plugging, which may manifest clinically as keratotic spikes revealed upon scale removal—the so-called “carpet tack sign”. Disease progression can lead to permanent follicular destruction and scarring alopecia. Over time, lesions become atrophic, often showing peripheral hyperpigmentation with central depigmentation, leading to irreversible, disfiguring skin changes [12].

Diagnosis is primarily clinical but is supported by histopathology and direct immunofluorescence in atypical or refractory cases. Histologically, DLE is characterized by hyperkeratosis, interface dermatitis with lymphocytic infiltrates, and basement membrane thickening. Direct immunofluorescence shows deposition of immunoglobulins or complement at the dermoepidermal junction in 50–90% of cases [13]. Treatment typically involves rigorous photoprotection and topical corticosteroids, with antimalarials and systemic corticosteroids reserved for more severe or treatment-resistant disease [9].

Although DLE is traditionally considered a skin-limited condition, emerging evidence suggests a capacity for systemic autoimmune activity. One important example is the development of antiphospholipid syndrome (APS).

Several case reports have documented APS arising in patients with longstanding DLE, even in the absence of systemic lupus erythematosus (SLE). A striking case from 2022 described a female patient who developed venous thrombosis and serologic positivity for antiphospholipid antibodies 12 years after the onset of dermatologic symptoms [15]. This observation aligns with findings from a case–control study showing elevated anticardiolipin antibody levels in patients with DLE compared to healthy controls. In contrast, a cross-sectional study reported a low prevalence of antiphospholipid antibodies, comparable to that in the general population.

These findings support the hypothesis that DLE, although clinically cutaneous, may be immunologically active systemically, potentially predisposing patients to complications like APS [11]. In the present case, such a complication reinforces the diagnosis of DLE as more than a skin-limited disease.

Due to its ability to mimic a wide range of dermatological and infectious conditions, discoid lupus erythematosus (DLE) is often referred to as “The Great Imitator”, amid many other conditions such as syphilis, tuberculosis, sarcoidosis, and a myriad of tumors, the differential diagnosis of which and the potential for imitation of other conditions is quite broad. This clinical mimicry significantly complicates the diagnostic process and necessitates the consideration of a broad differential diagnosis [16]. In the present case, several possibilities were considered, including fungal and bacterial infections, demodicosis, and cutaneous tuberculosis—each with its own characteristic yet overlapping clinical features [17].

Tinea faciei is a superficial dermatophyte infection that typically presents with centrifugally expanding, erythematous, scaly lesions with raised borders. Lesions often involve the cheeks, eyelids, or submandibular area and are usually pruritic. The differential diagnosis should include impetigo, atopic dermatitis, contact dermatitis, discoid lupus erythematosus, and herpes zoster [1]. In our case, lack of improvement despite antifungal treatment made this diagnosis unlikely [1].

Another diagnosis that was ruled out was bacterial skin infection (impetigo), which is most commonly caused by Staphylococcus aureus and Streptococcus pyogenes. Impetigo may present with erosions, vesicles, or pustules covered in honey-colored crusts. Bullous forms may exhibit large, thin-walled blisters. Multiple areas may be involved, and a combination of bullous and non-bullous lesions can be present simultaneously [6]. Negative bacterial cultures and lack of therapeutic response excluded this possibility.

Demodicosis was another suspected disease in the young patient. Caused by Demodex folliculorum, this condition can mimic rosacea, folliculitis, and other facial dermatoses. Three subtypes are recognized: pityriasis folliculorum, rosacea-like demodicosis, and granulomatous demodicosis. Presentation with atypical signs and symptoms that deviate from the classical forms is not uncommon [2]. Despite positive microscopic detection of Demodex, the lack of improvement following metronidazole therapy suggested it was not the primary cause.

Cutaneous tuberculosis (CTB), though rare (1–1.5% of all extrapulmonary TB cases), remains an important diagnostic consideration in chronic, non-healing facial plaques [5]. CTB includes entities such as lupus vulgaris, scrofuloderma, and tuberculosis verrucosa cutis. Historically, the term “lupus” originated in the disfiguring ulcerative lesions of facial cutaneous tuberculosis, described by Robert Willan.

In our patient, clinical similarities and a borderline-positive Mantoux test initially raised suspicion for cutaneous tuberculosis. However, histopathological analysis ultimately excluded the diagnosis. Nevertheless, tuberculosis warrants attention not only as part of the differential diagnosis but also as a potential secondary complication in immunosuppressed individuals. In patients with DLE receiving systemic immunomodulatory therapy, the risk of latent tuberculosis reactivation represents a clinically relevant concern. In the present case, this risk manifested as active tuberculosis that emerged during the course of treatment. The presence of radiologically confirmed foci of fibrosis and flares, together with the PCR-proven mycobacterial infection, would indicate that the aggressive immunomodulatory treatment, based on the significance, duration, and flares of DLE in the patient, led to reactivation of latent tuberculosis rather than a new-onset infection.

Immunosuppressive therapy is the cornerstone of treatment for all forms of lupus. While in most cases of cutaneous lupus, patients respond well to topical corticosteroid treatment, in cases of severe, especially long-lasting reactions, systemic administration is required [22]. As seen in our case, the patient initially responded well to combined systemic and local treatment; however, reducing the systemic corticosteroid dose led to new disease flares, necessitating prolonged, low-dose systemic administration. This treatment is closely linked to the pathogenesis of the condition because discoid lupus, despite being a limited cutaneous form, shares many immunological mechanisms with SLE and, although rare, can progress to SLE [22].

While broadly following the immunological mechanisms of SLE as an autoimmune reaction, the underlying cause is possibly that of keratinocyte nuclear damage. This mechanism is supported by the association of the condition with photosensitivity, in which UV light damages keratinocyte nuclei, and fragments of these nuclei can, in turn, stimulate an immune response [23,24]. These mechanisms would also explain the typical location of DLE, predominantly in the facial area—a topographical zone with significant sunlight exposure [22]. Alternatively, another nucleus-damaging factor—tobacco smoking—is also closely linked as a triggering factor for DLE. The site-specific immune response of B cells and possibly T cells, as well, based on these triggering factors, can explain some of the aspects that distinguish CCLE from SLE forms, and could underline the nature of DLE as a local reaction to external factors in patients with a genetic predisposition to SLE [22]. This would also explain the relatively low rate of progression to SLE and the co-occurrence of other immunological conditions, such as APS, as seen in our patient [15,22].

## 4. Conclusions

The presented findings emphasize the need for heightened clinical vigilance in patients with DLE, particularly in the presence of risk factors or nonspecific systemic symptoms. Monitoring antiphospholipid serological status in such individuals may aid in the early diagnosis and prevention of potential thrombotic complications. Based on the facts outlined above, it is reasonable to conclude that cutaneous lupus erythematosus can manifest with diverse characteristics, fully justifying its designation as one of the conditions bearing the moniker “The Great Imitator.”

## Figures and Tables

**Table 1:** Dynamics of complete and differential blood parameters.

	Leukocytes (WBC)	Neutro	Monocytes	Lymphocytes	HGB	Erythrocytes	ESR
Reference values	4.05–11.84 × 10^9^/L	2.07–7.73 × 10^9^/L	0.20–0.65 × 10^9^/L	1.17–3.45 × 10^9^/L	120–156 g/L	4.01–5.29 × 10^12^/L	2–37 mm/h
Initial presentation	4.62	3.29	0.17	0.95	95	2.96	93
Workup	12.11	10.25	0.32	1.31	101	3.13	41
Discharge	4.77	3.96	0.11	0.63	79	2.38	–

**Table 2:** Laboratory investigations revealing antiphospholipid syndrome.

Parameter	Patient’s Result	Reference Values
Quantitative determination of complement C3 fraction	1.662 g/L	0.81–1.57
Quantitative determination of complement C4 fraction	0.514 g/L	0.13–0.39
Antiphospholipid antibodies—ELISA—Anticardiolipin IgM	9.7 [MPL’U]/mL	0–7
Antiphospholipid antibodies—ELISA—Beta 2-glycoprotein-1 IgM	12.7 [arb’U]/mL	0–5

## Data Availability

Data regarding the study is freely available upon reasonable request from the authors.
